# Lesion Detection in Optical Coherence Tomography with Transformer-Enhanced Detector

**DOI:** 10.3390/jimaging9110244

**Published:** 2023-11-07

**Authors:** Hanya Ahmed, Qianni Zhang, Ferranti Wong, Robert Donnan, Akram Alomainy

**Affiliations:** 1 Department of Electronic Engineering and Computer Science, Queen Mary University of London—QMUL, London E1 4NS, UKr.donnan@qmul.ac.uk (R.D.); a.alomainy@qmul.ac.uk (A.A.); 2 Institute of Dentistry at Barts Health, Queen Mary University of London—QMUL, London E1 4NS, UK

**Keywords:** transformers, deep learning, image detection, optical coherence tomography, dentistry, computed tomography

## Abstract

Optical coherence tomography (OCT) is an emerging imaging tool in healthcare with common applications in ophthalmology for the detection of retinal diseases and in dentistry for the early detection of tooth decay. Speckle noise is ubiquitous in OCT images, which can hinder diagnosis by clinicians. In this paper, a region-based, deep learning framework for the detection of anomalies is proposed for OCT-acquired images. The core of the framework is Transformer-Enhanced Detection (TED), which includes attention gates (AGs) to ensure focus is placed on the foreground while identifying and removing noise artifacts as anomalies. TED was designed to detect the different types of anomalies commonly present in OCT images for diagnostic purposes and thus aid clinical interpretation. Extensive quantitative evaluations were performed to measure the performance of TED against current, widely known, deep learning detection algorithms. Three different datasets were tested: two dental and one CT (hosting scans of lung nodules, livers, etc.). The results showed that the approach verifiably detected tooth decay and numerous lesions across two modalities, achieving superior performance compared to several well-known algorithms. The proposed method improved the accuracy of detection by 16–22% and the Intersection over Union (IOU) by 10% for both dentistry datasets. For the CT dataset, the performance metrics were similarly improved by 9% and 20%, respectively.

## 1. Introduction

Optical coherence tomography (OCT) is an emerging medical imaging technique that uses low-coherence infrared light to harmlessly probe into the human body [1]. Low coherence, however, leads to speckle noise in imaging; thus, it gives rise to a poor signal-to-noise ratio (SNR), confounding imaging details and introducing artifacts [2]. OCT has been widely implemented in clinical practice for ophthalmology to detect multiple retinal diseases such as diabetic retinopathy (DR) [3] and age-related macular degeneration (AMD) [4]. Within preliminary dentistry research, OCT has been used for the early detection of carious lesions, but no procedures exist yet for adequately detecting tooth decay. With the advances in deep learning techniques applied to medical image analysis, it is possible to develop automated solutions that detect the disease in OCT imagery and provide quantitative evidence to medical experts. However, speckle noise in OCT presents a great challenge as it hinders clinicians’ ability to diagnose in extensive detail as well as the capability of deep learning models in their detection tasks. In previous decades, numerous image denoising approaches have been created, but they have proved inadequate for removing noise artifacts. Therefore, lesion detection methods have been developed detection in noisy OCT images. Nevertheless, there is not yet an adequate framework to overcome both denoising and lesion detection in OCT images.

Machine learning (ML) models have been utilized for image analysis, including detection, segmentation, and classification, within the medical field over the past decade. Focusing on lesion detection, the main concepts applied have been detection through classification or segmentation but not applying bounding boxes where the lesion is located. Lately, two main ML models have been successfully and widely implemented to output bounding boxes. These are You Only Look Once (YOLO) and Mask-R CNN (MRCNN). However, multiple drawbacks have been presented, including that YOLO struggles with generalizing anomalies in the form of new or uncommon aspect ratios or arrangements. Another drawback is the computational time and power taken for training and testing on new large datasets, which are not clinically efficient.

To address this problem, we propose a computational detection framework based on a Transformer-Enhanced Detection (TED) method with the assistance of denoising [5] It includes a special layout that combines transformers with attention gates (AGs) to suppress the loss of useful data and focus on the different types of anomalies in OCT images. A systematic comparison was conducted with existing state-of-the-art OCT detection algorithms to demonstrate the advantages of TED for clinical practice in multiple medical domains.

In summary, the contributions of this paper are as follows:

- A novel TED framework is developed, focusing on detecting relevant lesions in noisy OCT images of different organs.
- In TED, the transformer is adapted to take in images and slide across Regions of Interest (ROIs) provided by AGs. This design aims to adaptively deal with different types of noise artifacts and thus effectively detect a variety of anomalies including tooth decay and numerous lesions across two modalities.
- A new loss function is proposed along with TED, which combines a sliding box, Intersection Over Union (IOU), and Mean Squared Error (MSE). It compares the IOU and MSE between the predicted and real bounding boxes to evaluate the regions of focus chosen by the AGs.

### 1.1. Related Work

#### 1.1.1. Detection Methods

Over the past decade, machine learning (ML) models have been utilized for object detection, classification, and segmentation within the medical field [6]. They have provided effective and promising results for pulmonary lesion detection via pulmonary computed tomography (CT) scans, COVID-19 segmentation and diagnosis via X-rays, and many more applications [7,8]. Focusing on OCT image analysis, many convolutional neural networks (CNNs) have been implemented for the classification of retinal diseases such as diabetic retinopathy (DR) and age-related macular degeneration (AMD) [9,10]. In dentistry, they have been applied for caries detection via the transfer learning models VGG16 and ResNet50 or multi-layer perceptron (MLP) models [11,12,13]. These studies have shown relatively high accuracy, sensitivity, and specificity values of above 80%. However, the classification of images does not show the areas that are affected or indicate lesions. This does not aid clinicians in arriving at a faster diagnosis or improve classification where noise artifacts obscure or mislead [14]. A wide range of data is thereby prone to being mislabeled [14]. Researchers have therefore utilized U-Nets and autoencoders for the 2D segmentation of retinal and dental layers and V-Nets for the 3D segmentation of OCT volumes [15,16,17]. Segmentation provided similarly high efficiency through improved accuracy, yet most studies utilized denoised images as inputs rather than noisy images [14]. This is quite impracticable for clinical practice since there is yet to be an agreed denoising method for OCT imaging. Owing to this, many drawbacks of traditional denoising methods revolve around the loss of meaningful detail by their application of smoothing or the limited removal of noise. Additionally, most of these techniques are computationally intensive [14]. Lastly, another computer-aided diagnostic method is object detection, employing either one-stage or two-stage object identifiers. These include You YOLO and recurrent CNNs (R-CNNs), respectively [18,19]. These models apply bounding boxes where lesions are detected. YOLO has produced substantial results owing to the sliding-box feature it implements. The sliding box provides a convolution feature extractor that further provides confidence scores regarding which boxes contain the anomaly [20]. Numerous researchers have proved that YOLO is a suitable architecture for detection, but it struggles with generalizing anomalies in the form of new or uncommon aspect ratios or arrangements [20].

#### 1.1.2. Attention Gates and Transformers

In 2017, Bahdanau et al. first suggested an improvement from long-short term memory (LSTM), which is called attention gates (AGs) [21]. AGs was predominantly applied to natural language processing (NLP) and then later adjusted for computer vision [22]. Within computer vision, AGs are implemented in CNNs to ensure focus on a certain region to allow optimal feature extraction for the classification goal. Throughout training, AGs propose and underline fundamental Regions of Interest (ROIs) and subdue irrelevant background feature activations. Within medical image processing, the main aim of AGs is to perform the segmentation of lesions in image modalities such as breast ultrasound (BUS) [22]. Concentrating on OCT images, segmentation was used for specific diseases such as AMD and DR in OCT images [23]. All studies mentioned have only applied Tversky loss for segmenting images. Yet it has not been successfully applied for object detection, since its main objectives revolve around segmentation and classification.

Later, Dosovitskiy et al. in 2021 further refined AGs and produced attention-based ‘transformers’ that learn at a high effective rate, feature representations, from encoding long-range dependencies [24]. Transformers utilize a ‘multi-head’ attention model to associate long- and short-distance words in both forward and backward directions. Therefore, it outputs positional encoding for any sentence input [24]. Multiple previous research studies noticed the knowledge gap of transformers in computer vision. Therefore, Vision Transformers (ViTs) were developed to replace CNNs [25]. With regard to the medical field, ViT has been widely implemented for MRI, CT and X-rays for image classification, segmentation and reconstruction [25,26,27]. There is a knowledge gap for object detection of tooth decay using OCT images and ViT with effective and relatively accurate results.

## 2. Materials and Methods

**Figure 1:**
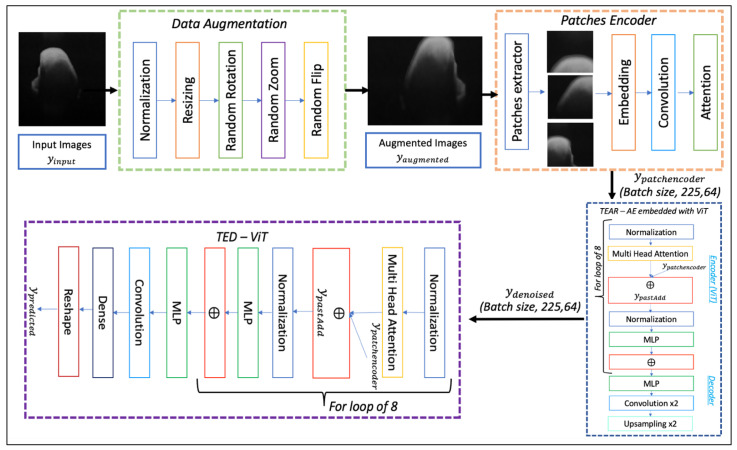
The architecture of the Transformer-Enhanced Detection (TED) framework. The denoised images are first augmented to create a larger dataset. The augmented images are then fed into the TED structure that contains a Vision Transformer (ViT) that is managed by the loss function during training.

In this paper, the proposed framework for OCT disease detection is described in Figure 1. Initially, data preparation is conducted, then augmentation is used to create more image/bounding pairs of boxes. This is to overcome the main disadvantage of limited clean OCT datasets for the training and validation of a model. The images are also denoised with an advanced ML method called TEAR where needed. The data are then input to a modified transformer that generates predicted outcomes for lesion detection with the help of the proposed $L_{Loss}$ loss.

### 2.1. Data Preparation and Augmentation

To ensure a fast and light-weight training, all OCT images are first resized to 250 pixels × 250 pixels. This is displayed in Figure 1 within the Data Augmentation block. The resized images are then augmented with rotation, magnification and inversion to create more image-bounding boxes to improve the training variety and quality. The resized images are flipped about the horizontal axes, using random rotation with a factor of 0.02 and random zoom in both width and height with a common factor of 0.2. Alongside augmentation, patches with a size of 32 pixels × 32 pixels are created from augmented images and passed through an AG.

### 2.2. Attention Gated Patch Encoder

As illustrated in Figure 1, within the patch encoder, AGs are placed to provide scores from the input, depending on ROIs that have the possible anomalies through the application of a sliding box. This is to aid the network in focusing on foreground detail and ignoring background information based on the content of each image. The patch encoder commences with a few layers that take the raw image $\left(I_{input}\right)$ as the first input, and the second input is set with the outputs of a sliding box. The outputs are patch sizes represented as a query value $\left(I_{queries}\right)$ (Equations (1) and (2)). Here, *Q* is a patch matrix, while *K* and *V* are key-value image pairs. Embedding layers (E) then process both inputs and convert each patch size into fixed-length vectors of defined size. The embedding layers utilize 64 × 64 units followed by convolution layers (∗) of 3 × 3 with filter size 32 and 64 with a stride of 3 (Equation (3)). It ensures that previous patches are taken into consideration with emphasis focused on areas through the input images and query values to compute matching scores. Weight vectors (WS) are created from the score, which are further processed through convolution layers, to calculate the matrices of both query and input patch size. Lastly, the convolution outputs are processed into a single-head attention layer. The number of convolution units is considered as a hyperparameter in all experiments, and it has been tuned accordingly. All the layers mentioned above are trainable and subject to change and adjustment for different types of medical images.

(1) $$I_{input}\left(Q,K,V\right)=\sum_{n=1}^{N}softmax\left(QK\right)\times V$$

(2) $$I_{queries}\left(Q,K,V\right)=\sum_{n=1}^{N}softmax\left(QK\right)\times V$$

(3) $$WS=E\left(A_{input}\right)*E\left(A_{queries}\right)$$

### 2.3. Transformer-Enhanced Detection

OCT is an emerging medical imaging modality; however, it is yet to be widely implemented beyond ophthalmology at present. Therefore, the main aim of TED focuses on aiding the detection of anomalies, including lesions and diseases, to simplify the diagnostic procedure for clinicians. As previously mentioned, ViTs are commonly considered as medical imaging processing tools for segmentation and classification tasks. They have yet to be adapted and implemented for object detection in medical images. Hence, the full framework after the patch encoder starts with TEAR. It consists of modifying ViT and placing it as an encoder in the autoencoder for denoising OCT images. TED follows and includes modifying ViT to output possible bounding boxes, indicating the location and label of relevant lesions. Within the TED framework, the original ViT procedure is followed [24], while reshaping the output of the patch encoder $y_{denoised}$ (TEAR output, Equation (4)) and flattening the patches to 2D images $y_{denoised}$∈RN×(P2·C). Here, (P,P) is the resolution of each image patch, *C* is the number of channels, *D* is the latent vector size of all the layers, *E* is the patch-embedding outputs, and *N* is the resulting number of patches (Equations (5) and (6)). The input is then submitted to multiple transformer blocks, starting with a normalization layer for the computation of mean and variance along all axes of the encoded patches $\left(z_{L}^{\prime }\right)$. Thus,

(4) $$y_{denoised}=TEAR\left(PE\left(DA\left(y_{input}\right)\right)\right)$$

(5) $$z_{0}=\left[y_{denoised};y_{p}^{1}E;y_{p}^{2}E;\cdots ;y_{p}^{N}E\right]+E_{pos}$$

(6) $$E\in R^{(P^{2}\cdot C)\times D},\;\;E_{pos}\in R^{(N+1)\times D}$$

(7) $$z_{l}^{\prime }=MSA\left(LN\left(z_{l-1}\right)\right)+z_{l-1},\;\;l=1\ldots L$$

(8) $$z_{l}=MLP\left(LN\left(z_{l}^{\prime }\right)\right)+z_{l}^{\prime },\;\;l=1\ldots L$$

(9) $$y_{predicted}=RS\left(LN\left(z_{l}^{\prime }\right)\right)$$

Equation (7) shows the encoded patches set to output the same dimension as the query dimension of the ‘multi-head’ attention (MSA) layer. Afterwards, we ensure that for each flattened input, $y_{predicted}$ is computed completely separately from other input features (Equation (9)). Next, the normalization layer (LN) outputs are passed onto a ‘multi-head’ attention layer for the computation of attention weights from similarity between two patches, as illustrated in Figure 1. Multiple heads process this in parallel, concatenating the cumulative result for one projected output. Equation (8) shows the projected output passed through another normalization layer that computes the mean and variance along the height, channels and width axes of images and then a multi-layer perceptron (MLP) block. The computed input features ensure that it is completely independent of other input features of other images in a batch. The MLP block acts as a classification head with Gaussian Error Linear Units (GELUs) featuring non-linearity [28]. The transformer block is repeated eight times, as indicated in Figure 1, which is followed by another MLP block for the final encoding of the image. Lastly, $y_{predicted}$ is reshaped (RS) into an array of possible bounding boxes (Equation (9)).

**Figure 2:**
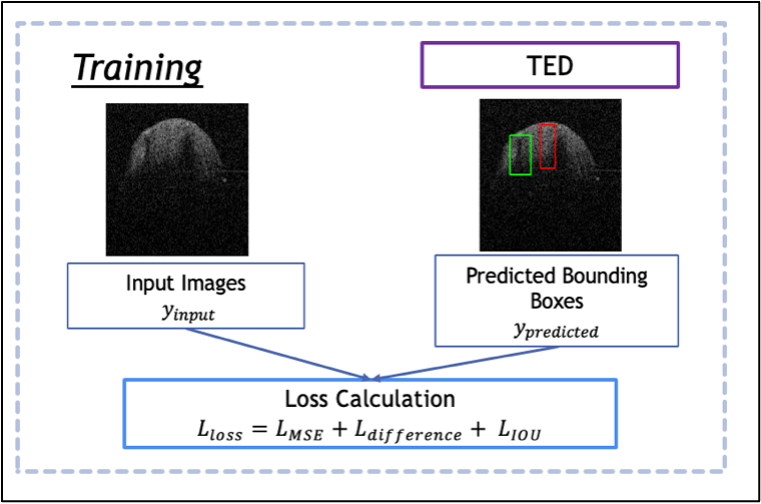
Training of Transformer-Enhanced Detection (TED) with the new proposed loss function, consisting of the combination of relevant detection evaluation metrics (MSE and IOU) between an input image and the predicted bounding box computed from TED. Here, the red and green boxes are the predicted and actual bounding boxes.

ViT is implemented after the patch encoder shown in Figure 1, in which it re-assembles an array of bounding boxes. The model in the framework is evaluated with numerous learning rates $\left(5.0\times 10^{-3},1.0\times 10^{-3},1.0\times 10^{-4},1.0\times 10^{-1},1.0\times 10^{-2}\right)$; epochs (200, 500 and 1000); batch sizes (2 and 4); optimizers (ADAM, ADAMW); and image sizes (500 pixels × 400 pixels, 500 pixels × 500 pixels). The TED model is trained through the process displayed in Figure 2, which utilizes a new proposed loss function. The environment in which the tests were implemented was Tensorflow and Keras, and all models were trained using one NVIDIA P100 GPU with 24 GB memory.

### 2.4. Loss Function

A new loss function is proposed for the ViT that is a combination of multiple quantitative metrics, where

(10) $$L_{Loss}=L_{MSE}+L_{difference}+L_{IOU}.$$

$L_{MSE}$ is the Mean Square Error loss calculated while training to provide the averaged batch size error of the $y_{predicted}$ and $y_{actual}$; $L_{difference}$ is the structural similarity difference loss between predicted and actual image pairs; and $L_{IOU}$ is the Intersection Over Union difference between actual and predicted IOU, which is a normalized coefficient [0, 1]. These are calculated as follows:

(11) $$L_{MSE}=\frac{1}{N}\sum_{i=1}^{N}{\left(y_{predicted}-y_{actual}\right)}^{2}$$

(12) $$L_{IOU}=1-\frac{\left|A\cap B\right|}{\left|A\cup B\right|}$$

*N* is the batch size provided in training; $y_{predicted}$ is the predicted bounding box by the CNN and $y_{actual}$ is the actual bounding box. Equation (12) shows how IOU loss is calculated as a ratio of area-of-overlap over area-of-union. The loss metrics are combined and normalized. The loss function aids the training of the model with a focus on minimizing the distance between predicted and actual bounding boxes. This is conducted with the help of MSE and IOU image metrics to ensure $y_{predicted}$ covers the correct amount of lesion/disease and within a reasonable size.

## 3. Results

### 3.1. Datasets

Three datasets from two medical fields were used to train and test the framework; the two dentistry datasets were one collected in the QMUL IDIOT Lab (QueenMary University of London, Institute of Dentistry in which the free space SD-OCT was set up and utilized to scan the teeth and models) and an immense CT dataset called NIH DeepLesion [29]. Dentistry datasets are imaged by a spectral domain OCT (SD-OCT) with an axial resolution of 4.5 μm and consist of ten samples of 500 images of healthy and decayed teeth. Each image is 500 pixels × 412 pixels. Clean images were created for the dentistry dataset 1 by pre-processing it through TEAR [5] The NIH DeepLesion dataset contains 32, 120-axial CT slices from 10,594 studies. All images included in this paper have one to three lesions per image. Regarding the dentistry datasets, lesions were drawn by multiple dentists and further used as ground truth. Each dataset is then randomly split into training, validation and testing with a 60%:10%:30% split, respectively.

### 3.2. Evaluation Metrics

The proposed framework is examined using conventional detection metrics: IOU, accuracy and a confusion matrix for quantitative evaluation. A confusion matrix is populated with true positive (TP) elements (for which IOU > 0.5) and false positive (FP) elements (for which IOU < 0.5). The numerical metrics consist of accuracy, sensitivity, precision, F1 score and IOU. In these, TN is when the model predicts images as having no lesions correctly, and FN is when it predicts false lesions. Regarding confusion matrix calculations, true negative (TN) and false negative (FN) are set to zero since it is a detection framework with only one class set to identify lesions. Sensitivity is sometimes called true positive rate (TPR) or recall. Both are tests of the ability to identify predictions correctly. Precision is a ratio of accurate predictions against all positive predictions (both true and false). The F1 score is a better measure to provide a sustainable balance between precision and sensitivity as well as provide a metric if there is a class imbalance. IOU is an evaluation metric mainly utilized for measuring the accuracy of an object detector traditional program, i.e., a ratio of area-of-overlap over area-of-union.

### 3.3. Ablation Study

TED with different settings was evaluated in a study to investigate the optimal learning rates, epochs, batch, optimizer and image sizes. The batch size was set to four, and the optimal image size was 500 pixels × 400 pixels and 500 × 500 pixels. The leading learning-rate and epochs were $5\times 10^{-4}$ and 200, respectively, to obtain optimal results in a suitable timely manner.

**Figure 3:**
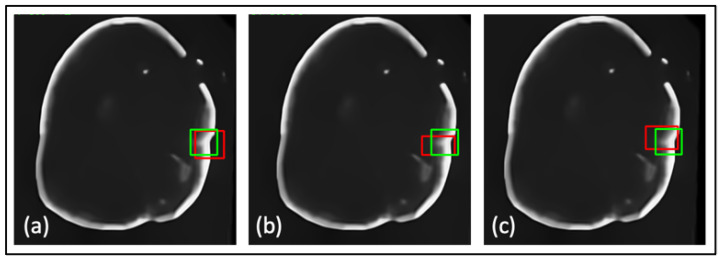
Results from comparative study for dentistry dataset 1 where (**a**) is the Transformer-Enhanced Detection method (TED with $L_{Loss}$), (**b**) is the MSE loss, and (**c**) is the MAE loss. Green boxes are actual bounding boxes ($y_{actual}$), and red boxes are predicted bounding boxes ($y_{predicted}$).

**Figure 4:**
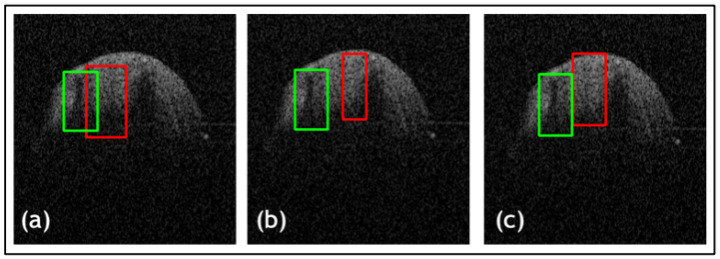
Results from comparative study for dentistry dataset 2 where (**a**) is the proposed method (TED with $L_{Loss}$), (**b**) is the MSE loss, and (**c**) is the MAE loss. Green boxes are actual bounding boxes ($y_{actual}$), and red boxes are predicted bounding boxes ($y_{predicted}$).

**Figure 5:**
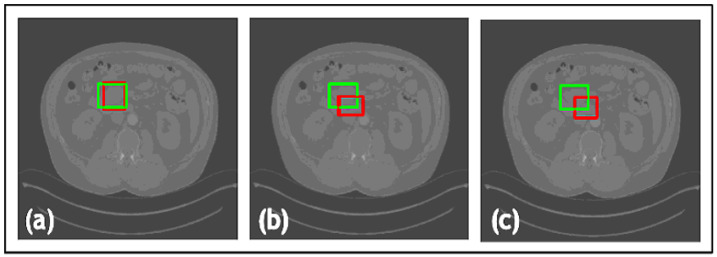
Results from the comparative study for NIH DeepLesion where (**a**) is the Transformer-Enhanced Detection method (TED with $L_{Loss}$), (**b**) is the MSE loss, and (**c**) is the MAE loss. Green boxes are actual bounding boxes ($y_{actual}$), and red boxes are predicted bounding boxes ($y_{predicted}$).

Furthermore, a comparative study was conducted on the datasets to evaluate the proposed loss function against widely used loss functions for object detection. The loss functions examined against $L_{Loss}$ consist of Mean Squared Error (MSE) and Mean Absolute Error (MAE). The quantitative evaluations are shown in Table 1 and the leading qualitative evaluation is shown in Figure 3, Figure 4 and Figure 5 for both the dentistry and CT datasets, respectively. The results are averaged over the dataset. The sensitivity is unity for all evaluation tests, since the confusion matrix only counts TP and FP for lesion detection; therefore, TN and FN are zero. Since the datasets utilized in this paper only have bounding boxes for lesions, therefore, there is only one class that is set as a lesion. Hence, accuracy and precision have equivalent metrics for all the tests. Quantitative results display that TED-$L_{Loss}$ performs 15–30% higher in average accuracy and precision, acquiring an average of 92.5% in both. The F1 score had an increase of 10–15%, demonstrating a more stable lesion detection with $L_{Loss}$ than MSE and MAE losses. Regarding IOU, TED obtains an average of increase of 25% in dentistry datasets. This is validated with qualitative results in Figure 3a and Figure 4a, in which $y_{predicted}$ (red boxes) overlap the majority of $y_{actual}$ (green boxes). Additionally, compared to $L_{Loss}$, MAE and MSE produced bounding boxes either too small or big, hence the drop in IOU. With regard to the CT dataset, quantitatively, TED-$L_{Loss}$ obtained the best-performing average accuracy, F1 score and IOU of 97%, 0.98 and 0.81, respectively. That is an increase of 10%, 18% and 15% across the evaluation metrics, respectively. Along with the qualitative results shown in Figure 5a, the best performing results in the test dataset had an IOU of 0.96, accurately overlapping $y_{actual}$ (green boxes). This is in contrast to MSE and MAE (Figure 5b,c), producing smaller $y_{predicted}$ (red boxes) that partially overlap $y_{actual}$ (green boxes). This demonstrates the value of implementing $L_{Loss}$ in TED for lesion detection across two image modalities. This is shown by the increase in all quantitative metrics for all datasets.

**Figure 6:**
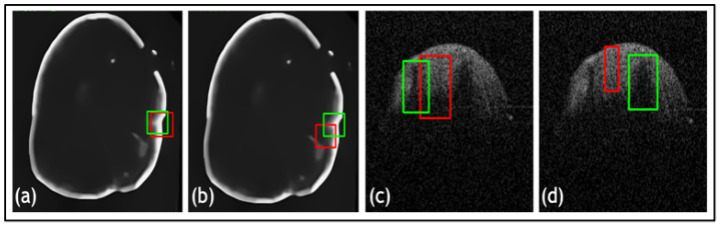
Results from the comparative study for dentistry datasets 1 and 2 where (**a**) is the Transformer-Enhanced Detection method (TED with AG), (**b**) is TED without AG for dataset 1, (**c**) is TED with AG, and (**d**) is TED without AG for dataset 2. Green boxes are actual bounding boxes ($y_{actual}$), and red boxes are predicted bounding boxes ($y_{predicted}$).

Another ablation study was used to compare the TED framework with and without the additional attention gates in the patch encoder and data augmentation. The quantitative evaluations are shown in Table 2 and leading qualitative evaluation in Figure 6, for both dentistry datasets, respectively. Quantitative results display that TED with AGs performs 9% higher on average accuracy and precision, acquiring an average of 92.5% in both. The F1 score had an increase of 8–18%, demonstrating a more stable lesion detection with AGs, than without. Regarding IOU, AGs provides an average of increase of 15% in both dentistry datasets. This is validated with the qualitative results in Figure 6a,c, in which $y_{predicted}$ (red boxes) overlap the majority of $y_{actual}$ (green boxes). Figure 6 presents that TED with AGs provides a preferable overlap between $y_{actual}$ and $y_{predicted}$ with a suitable bounding box size. This demonstrates the value for implementing AGs alongside $L_{Loss}$ in TED for lesion detection across both dentistry datasets. This is shown by the increase in all quantitative and qualitative metrics for all datasets.

### 3.4. Results and Comparison

**Figure 7:**
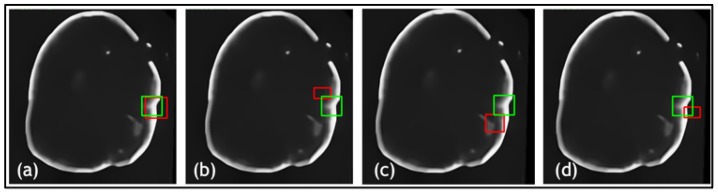
Results from comparative study for dentistry dataset 1 where (**a**) TED with $L_{Loss}$, (**b**) YOLOv1,b29, (**c**) YOLOv3 [31], (**d**) RCNN [32]. Green boxes are actual bounding boxes ($y_{actual}$) and red boxes are predicted bounding boxes ($y_{predicted}$).

**Figure 8:**
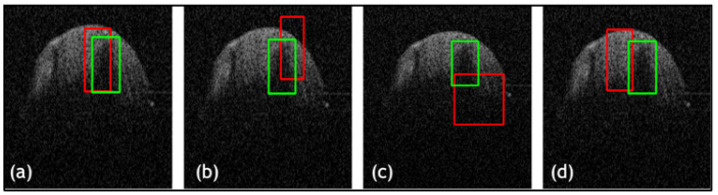
Results from comparative study for dentistry dataset 2 where (**a**) TED with $L_{Loss}$, (**b**) YOLOv1 [30], (**c**) YOLOv3 [31], and (**d**) RCNN [32]. Green boxes are actual bounding boxes ($y_{actual}$) and red boxes are predicted bounding boxes ($y_{predicted}$).

**Figure 9:**
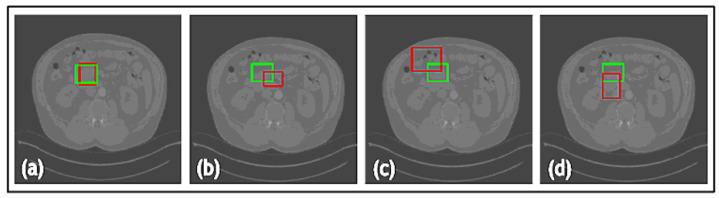
Results from comparative study for NIH DeepLesion dataset where (**a**) TED with $L_{Loss}$, (**b**) YOLOv1 [30], (**c**) YOLOv3 [31], and (**d**) RCNN [32]. Green boxes are actual bounding boxes ($y_{actual}$) and red boxes are predicted bounding boxes ($y_{predicted}$).

In addition, a comparative study was conducted on the three datasets to examine the proposed framework against state-of-the-art DL techniques YOLOv1 [30], YOLOv3 [31] and Mask-RCNN [32]. Quantitative results, with evaluation metrics for the TED method against state-of-the-art detectors for dentistry and CT datasets, are shown in Table 3. The results are averaged over the datasets. Numerous images are shown from each dataset for qualitative comparison of the outputs of the well-known detectors with respect to our proposed framework, as shown in Figure 7, Figure 8 and Figure 9. Quantitative results display that TED-$L_{Loss}$ performs 16–22% higher in average accuracy and precision, acquiring an average of 92.5% in both dentistry datasets. Mask-RCNN outperformed YOLOv1 and YOLOv3 in dentistry dataset 1 by obtaining an accuracy and average IOU of 76% and 0.79, respectively. This is displayed in Figure 7 in which Mask-RCNN covered a larger amount of the green box than YOLOv1 and YOLOv3. However, Mask-RCNN did not obtain the best quantitative results across dentistry dataset 2 and NIH DeepLesion. This is attributed to a clear misdiagnosis, as shown by the red boxes in Figure 8 and Figure 9 not overlapping with the green boxes in significant proportion. Across all datasets examined, YOLOv3 produced relatively larger $y_{predicted}$ (red boxes), as shown in Figure 7c, Figure 8c and Figure 9c. On the other hand, YOLOv1 (Figure 7b and Figure 9b) produced smaller $y_{predicted}$ (red boxes). This would complicate diagnosis for clinicians, especially for more complex images. With regard to the CT dataset, quantitatively, TED-$L_{Loss}$ obtained the leading metrics in average accuracy, F1 score and IOU with values of 97%, 0.98 and 0.81, respectively. This is an increase of 9%, 4% and 20% across the evaluation metrics, respectively, indicating the value for utilizing TED-$L_{Loss}$ for lesion detection across two image modalities. This is shown by the increase in all quantitative metrics for all datasets against well-known object detection CNNs.

Lastly, each DL model was timed for training and testing functionalities for the dentistry datasets and NIH DeepLesion, which consist of 5000 images of size 512 × 500 pixels and 32,120 images of size 512 × 512 pixels, respectively. This is to show and evaluate if they provide results within a timely manner, focusing more on the training time taken and for the adaptation of new and different datasets when required in an efficient process. Therefore, the results are displayed in Table 4 for the CNN models against TED-$L_{Loss}$ for the three datasets. Regarding dentistry datasets 1 and 2 and the NIH DeepLesion dataset, the proposed method was trained and tested within 9 min 51 s, 10 min 2 s and 3 min 60 s, respectively. This produces an average time of 7 min 57 s taken. That is at least less than 11% over other DL models. Therefore, it implies that TED-$L_{Loss}$ is a lightweight model that has numerous hyperparameters that are subject to training for different datasets.

## 4. Discussion

Recently, DL methods have been implemened for many image processing tasks, such as classification, segmentation and detection. Numerous CNN layouts were implemented for low-dose CT and MRI [33,34]. Nonetheless, there is limited research focused on lesion detection in SD-OCT datasets. The leading DL models are RCNN [32] and developments of YOLO (such as YOLOv1 [30], YOLOv3 [31]). These were implemented on public multimodality datasets, such as NIH DeepLesion [29]. DL methods were either not compared against other popular DL methods or proven to improve the next-step analysis of images. Next-step analysis examples include lesion classification as well as dental and abdomen layer segmentation. Furthermore, there is yet to be a DL model implemented for the lesion detection of SD-OCT dental images. Hence, the TED method here is the first work undertaken to show efficacy through comparison against DL detection methods and further analysis against assessment given by a dentist. A key task is to apply detection on more than one dataset of different medical imaging modalities. This was validated here by using both CT and OCT data libraries.

Three datasets were trained and tested: one was a public CT dataset [29] that consisted of CT slices with a table of bounding boxes of lesions in each image; the second and third are dental datasets that consisted of OCT images with a respective table of bounding boxes around lesions. Results show that the TED method generated superior detection capability over multiple modalities even with limited data of OCT images.

The TED method has several advantages that are novel. Firstly it deploys attention gates (AGs) into the data augmentation operation to provide the model with foreground ROIs. This allows the model to focus on creating a larger dataset from the limited data provided from either medical imaging modalities. Additionally, it is expected to aid the network to focus on the foreground and ignore background information based on the content of each image, which later provide specified patches for the CNN to denoise and detect accurately. This also allows the framework to utilize less computational power and time, since it replaces the concept of two function CNNs for feature extraction before medical image analysis.

After the AG model and data augmentation, the patches are fed into the TEAR framework for data preparation to create clean reference images in order to ensure the minimal amount of removal of useful data. TEAR is a new hybrid of ViT, which was implemented as an encoder in an autoencoder to utilize the attention score from AGs and correlate the ROIs to reconstruct the image in the correct manner. Specifically, this did not include the addition of data due to any realignment issue or noise artefact or limiting the removal of useful data between the OCT dental and CT abdominal layers. Instead, the proposed loss function focused on signal restoration, error in data retrieval, thresholding and edge preserving. This creates a robust framework, since different types of noise artefacts and speckle noise were removed to an appropriate limit without removing useful data.

Next, the denoised patches are passed to TED, which is a new hybrid of adapted ViT with additional AGs. An ablation study in Section 3.3, displayed the added value of AGs for detection. The quantitative evaluations are shown in Table 4 and the leading qualitative evaluation is shown in Figure 6 for both dentistry datasets, respectively. Quantitative results display that TED with AGs performs 9% higher in average accuracy and precision, acquiring an average of 92.5% in both. With regard to IOU, AGs provides an average of increase of 15% in both dentistry datasets. Another contribution shown in this papers is a newly implemented loss function, providing the multiple image quality metrics of a sliding box, IOU, and MSE. IOU focuses on bounding boxes placement and MSE in object detection. This creates a robust framework for different types of image modalities in numerous medical fields. In Section 3.3, a comparative study is performed to exhibit the performance of the loss function. Quantitative results display that TED-$L_{Loss}$ performs 15–30% higher in average accuracy and precision, acquiring an average of 92.5% in both. The F1 score had an increase of 10–15%, demonstrating a more stable lesion detection with $L_{Loss}$ than MSE and MAE losses. With regard to IOU, TED obtains an average increase of 25% in the dentistry datasets. With regard to the CT dataset, TED-$L_{Loss}$ obtained the best-performing average accuracy, F1 score and IOU of 97%, 0.98 and 0.81, respectively. That is an increase of 10%, 18% and 15% across the evaluation metrics, respectively.

**Figure 10:**
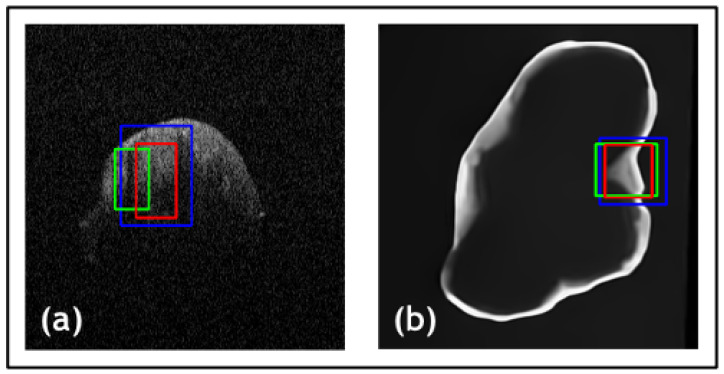
Results from the comparative study for dentistry datasets. (**a**) is dataset 1 and (**b**) is dataset 2. Green boxes are actual bounding boxes ($y_{actual}$), blue boxes are the dentists’ predicted bounding boxes, and red boxes are predicted bounding boxes ($y_{predicted}$).

Within this paper, dentists are queried as to whether the proposed method detects the lesion effectively for them to diagnose. For dentistry dataset 1 (Figure 10a), dentists acquired an average IOU of 0.87 against the actual bounding boxes in which the dentist and predicted boxes overlap completely. Moving onto dentistry dataset 2 (Figure 10b), all bounding boxes overlap in an ideal manner. This produces an average IOU of 0.97, acknowledging that the dentist’s bounding box is relatively larger while the predicted bounding box is narrower.

## 5. Conclusions

This paper introduces Transformer-Enhanced Detection (TED), which is a novel framework that detects lesions in two medical imaging modalities, optical coherence tomography (OCT) and computed tomography (CT). The OCT images were captured by Spectral Domain OCT (SD-OCT) for dentistry fields and CT over multiple fields. TED delivers substantial advantage to clinicians since it maintains useful information to aid in clear and unambiguous diagnosis. It starts with overcoming the first obstacle of employing OCT by supplementing the limitation of clean OCT datasets through data augmentation. This aids in optimizing the supervised learning within the architecture. The augmented data are then the argument to TED. The workings consist of a layout combining transformers with attention gates to decrease the loss of useful data and to promote focus on the different types of anomalies in OCT images. The new loss function is utilized along with TED, which combines a sliding box, Intersection Over Union (IOU) and Mean Squared Error (MSE). TED improved the accuracy of lesion detection by 16–22% and IOU by 10% in both dentistry datasets and by 9% and 20%, respectively, for the CT dataset. Through testing multiple datasets, it was demonstrated that the TED framework has the ability of being applied to different modalities across multiple medical fields. It has been shown that its framework is capable of automatically adapting to different datasets. This new detection methodology will be next conducted on magnetic resonance imaging (MRI) datasets and OCT cardiology datasets. Further work will also look to simulate the effect of further innovations of the precise segmentation of lesions.

## Figures and Tables

**Table 1:** Quantitative results of lesion detection task for dentistry datasets 1 and 2 and NIH DeepLesion dataset of proposed method with different loss functions regarding the averaged accuracy, precision, F1 score and IOU.

Net	Accuracy	Precision	F1 Score	IOU
Dentistry Dataset 1				
TED-MSE	0.72	0.72	0.76	0.66
TED-MAE	0.68	0.68	0.78	0.68
TED-$L_{Loss}$	0.89	0.89	0.84	0.83
Dentistry Dataset 2				
TED-MSE	0.80	0.80	0.84	0.81
TED-MAE	0.92	0.92	0.90	0.71
TED-$L_{Loss}$	0.96	0.96	0.98	0.84
NIH DeepLesion Dataset				
TED-MSE	0.87	0.87	0.83	0.70
TED-MAE	0.90	0.90	0.85	0.73
TED-$L_{Loss}$	0.97	0.97	0.98	0.81

**Table 2:** Quantitative results of lesion detection task for dentistry datasets 1 and 2 and NIH DeepLesion dataset of proposed method with and without attention gates (AGs) in averaged accuracy, precision, F1 score and IOU.

Net	Accuracy	Precision	F1 Score	IOU
Dentistry Dataset 1				
TED—without AG	0.82	0.82	0.78	0.73
TED—with AG	0.89	0.89	0.84	0.83
Dentistry Dataset 2				
TED—without AG	0.88	0.88	0.84	0.74
TED—with AG	0.96	0.96	0.98	0.84

**Table 3:** Quantitative results of lesion detection task for dentistry datasets 1 and 2 and NIH DeepLesion dataset of the proposed method against well-known CNNs in averaged accuracy, precision, F1 score and IOU.

Net	Accuracy	Precision	F1 Score	IOU
Dentistry Dataset 1				
YOLOv1 [30]	0.73	0.73	0.84	0.67
YOLOv3 [31]	0.74	0.74	0.85	0.69
Mask-RCNN [32]	0.76	0.76	0.86	0.79
TED-$L_{Loss}$	0.89	0.89	0.84	0.83
Dentistry Dataset 2				
YOLOv1 [30]	0.93	0.93	0.96	0.77
YOLOv3 [31]	0.87	0.87	0.93	0.79
Mask-RCNN [32]	0.74	0.74	0.86	0.73
TED-$L_{Loss}$	0.96	0.96	0.98	0.84
NIH DeepLesion Dataset				
YOLOv1 [30]	0.88	0.88	0.94	0.54
YOLOv3 [31]	0.94	0.94	0.96	0.61
Mask-RCNN [32]	0.71	0.71	0.83	0.53
TED-$L_{Loss}$	0.97	0.97	0.98	0.81

**Table 4:** Time taken of lesion detection task for training and testing on dentistry datasets 1 and 2 (5000 images of size 500 × 412 pixels) and NIH DeepLesion (32,120 images of size 512 × 512 pixels) of proposed method against well-known CNNs in minutes and seconds.

Net	Time Taken (Min s)
Dentistry Dataset 1	
YOLOv1 [30]	10 m 36 s
YOLOv3 [31]	20 m 3 s
Mask-RCNN [32]	9 m 25 s
TED-$L_{Loss}$	9 m 51 s
Dentistry Dataset 2	
YOLOv1 [30]	10 m 33 s
YOLOv3 [31]	20 m 13 s
Mask-RCNN [32]	10 m 46 s
TED-$L_{Loss}$	10 m 2 s
NIH DeepLesion Dataset	
YOLOv1 [30]	6 m 32 s
YOLOv3 [31]	7 m 44 s
Mask-RCNN [32]	4 m 25 s
TED-$L_{Loss}$	3 m 59 s

## Data Availability

All relevant code and data are available upon request.
